# Identification of Two Independent Risk Factors for Lupus within the MHC in United Kingdom Families

**DOI:** 10.1371/journal.pgen.0030192

**Published:** 2007-11-09

**Authors:** Michelle M. A Fernando, Christine R Stevens, Pardis C Sabeti, Emily C Walsh, Alasdair J. M McWhinnie, Anila Shah, Todd Green, John D Rioux, Timothy J Vyse

**Affiliations:** 1 Section of Molecular Genetics and Rheumatology, Imperial College London, London, United Kingdom; 2 The Broad Institute of MIT and Harvard, Cambridge, Massachusetts, United States of America; 3 Histocompatibility Laboratories and Research Institute, The Anthony Nolan Trust, London, United Kingdom; 4 Université de Montréal, Montréal Heart Institute, Montréal, Québec, Canada; North Carolina State University, United States of America

## Abstract

The association of the major histocompatibility complex (MHC) with SLE is well established yet the causal variants arising from this region remain to be identified, largely due to inadequate study design and the strong linkage disequilibrium demonstrated by genes across this locus. The majority of studies thus far have identified strong association with classical class II alleles, in particular HLA-DRB1*0301 and HLA-DRB1*1501. Additional associations have been reported with class III alleles; specifically, complement *C4* null alleles and a tumor necrosis factor promoter SNP (*TNF-308G/A*). However, the relative effects of these class II and class III variants have not been determined. We have thus used a family-based approach to map association signals across the MHC class II and class III regions in a cohort of 314 complete United Kingdom Caucasian SLE trios by typing tagging SNPs together with classical typing of the *HLA-DRB1* locus. Using TDT and conditional regression analyses, we have demonstrated the presence of two distinct and independent association signals in SLE: HLA-DRB1*0301 (nominal *p* = 4.9 × 10^−8^, permuted *p* < 0.0001, OR = 2.3) and the T allele of SNP *rs419788* (nominal *p* = 4.3 × 10^−8^, permuted *p* < 0.0001, OR = 2.0) in intron 6 of the class III region gene *SKIV2L*. Assessment of genotypic risk demonstrates a likely dominant model of inheritance for HLA-DRB1*0301, while *rs419788-T* confers susceptibility in an additive manner. Furthermore, by comparing transmitted and untransmitted parental chromosomes, we have delimited our class II signal to a 180 kb region encompassing the alleles HLA-DRB1*0301-HLA-DQA1*0501-HLA-DQB1*0201 alone. Our class III signal importantly excludes independent association at the *TNF* promoter polymorphism, *TNF-308G/A,* in our SLE cohort and provides a potentially novel locus for future genetic and functional studies.

## Author Summary

Systemic lupus erythematosus (SLE/lupus) is a complex autoimmune disease in which the body's immune system attacks its own tissues, causing inflammation in a variety of different organs such as the skin, joints, and kidneys. The cause of lupus is not known, but genes play a significant role in the predisposition to disease. The major histocompatibility complex (MHC) on Chromosome 6 contains at least 100 different genes that affect the immune system, including the genes with the strongest effect on lupus susceptibility. Despite the importance of the MHC in SLE, the identity of the actual genes in the MHC region that cause SLE has remained elusive. In the present study, we used the latest set of genetic markers present at the MHC in lupus families to identify the actual genes that affect the disease. To our knowledge, we have shown for the first time that two separate groups of genes are involved in SLE. One group of genes alters how the immune system may inappropriately target its own tissues in the disease. How the second set of genes predisposes to SLE is the subject of ongoing study.

## Introduction

Since the early 1970s, the human major histocompatibility complex (MHC) has been shown to be associated with a number of autoimmune, inflammatory, and infectious diseases, and it continues to be the focus of intense research [1]. The recently defined extended MHC (xMHC) encompasses 7.6 Mb of genome on 6p21.3 and is divided into five subregions from telomere to centromere: extended class I, classical class I, classical class III, classical class II, and extended class II. In addition, the MHC contains two hypervariable regions, the RCCX module in class III (spanning complement *C4*) and the *HLA-DRB* genes in class II, that both exhibit copy number polymorphism. Examination of the sequence across the extended MHC reveals the presence of 421 genes, and over 252 (60%) are thought to be expressed [2]. Around 40% of genes expressed within the classical MHC encode proteins with putative immunomodulatory function [3]. The classical class I and class II loci encode the human leucocyte antigen (HLA) proteins involved in antigen presentation to T cells, initiating the adaptive immune response. The class III region contains the greatest density of genes in the genome (58 expressed genes), which are often found in functionally related clusters [2].

A major obstacle in the identification of disease-specific causal variants within the MHC has been the strong linkage disequilibrium (LD) exhibited by certain alleles in this region, resulting in the existence of long-range, conserved, extended haplotypes [4], also known as ancestral haplotypes [5], sometimes spanning more than 2 Mb [6]. Thus, for many MHC-associated diseases, it has only been possible to delimit association signals to a particular extended haplotype or segment of one.

Systemic lupus erythematosus (SLE/lupus, [Online Mendelian Inheritance in Man 152700, http://www.ncbi.nlm.nih.gov/sites/entrez?db=OMIM&TabCmd=Limits]) is a chronic, multi-system autoimmune disease affecting young women ten times more commonly than men. The worldwide prevalence of SLE is estimated at between 12 and 124 cases per 100,000 individuals [7]. SLE is characterized by the presence of pathogenic autoantibodies to nuclear and cell-surface antigens that show affinity maturation. The consequent immune complexes deposit in tissues, causing inflammation and damage. It is well established that there is a complex genetic component to lupus aetiology, with hormonal and environmental influences also contributing to disease susceptibility [8,9].

The MHC has been the most consistently confirmed genetic risk factor for SLE, and multiple different genes within the region have been significantly implicated with disease susceptibility. For example, hereditary and acquired deficiencies of the early classical complement component C4, located within the MHC class III locus, leads to a lupus-like syndrome. A role for another class III gene, tumour necrosis factor alpha (*TNF*), in SLE was suggested following the observation that the lupus-prone New Zealand F1 mouse hybrid exhibits constitutively low TNF expression [10]. Recently, the development of antinuclear antibodies in patients treated with TNF antagonists has also stimulated interest in the possible role of TNF in SLE [11–13]. Murine and human candidate gene studies, together with genome-wide linkage screens, provide further support that multiple genetic loci, including the mouse MHC complex *H2* and the human MHC locus, contribute to disease susceptibility [14–17].

It should be noted that the human MHC was first associated with SLE in 1971, when studies demonstrated that lupus probands were enriched for the class I alleles *HL-A8* (now known as *HLA-B8*) and *HLA-W15* (now known as *HLA-B15*) when compared with healthy controls [18,19]. Further case control association studies were small, performed in ethnically diverse populations, and tested only a small number of the classical HLA and complement *C4* alleles. The most consistent findings reported to date are associations with the class II alleles *HLA-DR2* (DRB1*1501) and *HLA-DR3* (DRB1*0301) and their respective haplotypes in Caucasian populations. The complement *C4A* null allele (C4A*Q0) has shown inconsistent association with lupus in a number of studies—a situation that may reflect genetic heterogeneity in disease susceptibility [20–23]. In addition, a recent study has demonstrated that low *C4A* copy number is a risk factor for lupus in a European American cohort [24]. However, the *C4A* null allele lies on the lupus-associated DR3 “autoimmune” extended haplotype (AH8.1), which exhibits extremely strong LD [6]. It therefore remains to be definitively established whether this locus constitutes a distinct susceptibility allele to that of the class II association or is merely in LD with it. Similarly, certain *TNF* promoter polymorphisms, including the much-studied SNP *TNF-308G/A,* have shown association with SLE; but again, many of these variants are carried on the highly conserved 8.1 ancestral haplotype, thus restricting interpretation of these data.

In 2002, a family-based study employing microsatellites as surrogate markers for *HLA-DRB1* haplotypes in Caucasian lupus families demonstrated association with *DR3-*, *DR2-*, and *DR8* (DRB1*0801)-containing haplotypes. In that study, Graham and colleagues reported that, taking advantage of recombinant chromosomes, the disease risk region could be limited to a 1 Mb region encompassing classical class II and class III [25].

We have performed a medium resolution association mapping study of the MHC in lupus families, utilizing a combination of SNPs and four-digit typing at the *HLA-DRB1* locus in order to anchor haplotypes. Sixty-eight SNPs were successfully genotyped across a 2.4 Mb region of the MHC, from the class I locus *KIAA1949* to the class II gene *HLA-DPB2*, in 314 UK Caucasian SLE trios. We used these data to perform a family-based association study in an attempt to distinguish the relative effects of the class II and class III regions of the MHC in lupus susceptibility. In addition, we employed the long-range haplotype test to search for the presence of high-frequency, extended haplotypes indicative of recent positive selection [26]. We have also used family-based and case-control strategies to examine genotypic risk at *HLA-DRB1* and *rs419788*.

## Results

### Association Testing of *HLA-DRB1* and MHC Region SNPs

In order to define the causal variation within the MHC region, we typed 314 complete SLE trios for the *HLA-DRB1* gene as well as for 86 SNPs across a 2.4 Mb region encompassing the HLA class I locus *HLA-B* to *HLA-DPB2*. High-quality genotype data was obtained for *HLA-DRB1* and 68 MHC SNPs (see Table S1 for quality control data). Association testing of the *HLA-DRB1* gene revealed a significant association with HLA-DRB1*0301 (nominal *p* = 4.9 × 10^−8^, permuted *p* < 0.0001, T:U = 129:55) in our lupus cohort (Table 1). There was also a trend for under transmission of the HLA-DRB1*0701 allele (nominal *p* = 0.0013, T:U 42:77); however, this association was no longer significant after correction for multiple testing as determined by 10,000 permutations of the dataset (permuted *p* = 0.09). Furthermore, we did not find evidence of association with HLA-DRB1*1501 (nominal *p* = 1.0, T:U 70:70) or HLA-DRB1*0801 (nominal *p* = 1.0, T:U = 11:11) in our cohort (see Table S2 for complete *HLA-DRB1* association data); alleles previously suggested by microsatellite typing of a US lupus cohort [25].

**Table 1:**
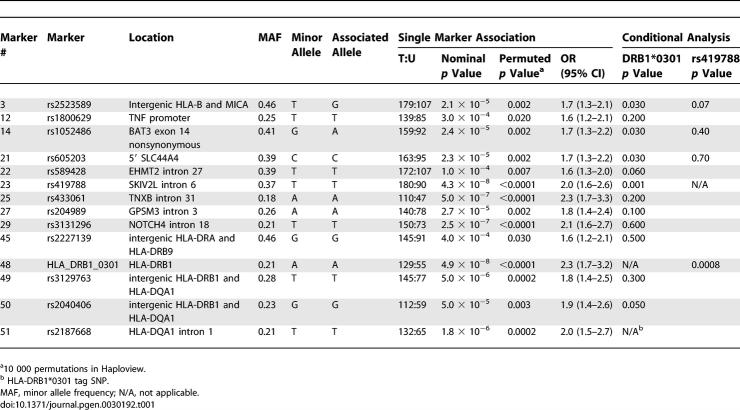
Single Marker Association and Conditional Regression Analysis in 314 UK SLE Trios

**Figure 1:**
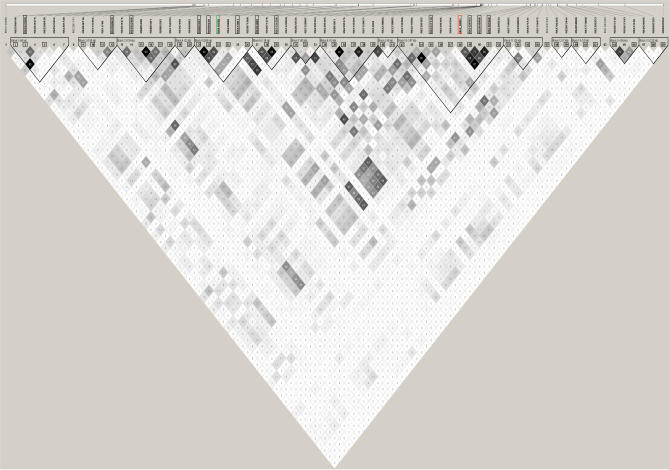
LD Plot of All 69 MHC Markers Typed in 314 UK SLE Trios The figure illustrates the correlation between all 69 MHC markers successfully typed across 2.4 Mb of genome encompassing MHC class II and class III in 314 UK SLE trios. The markers are identified by rs numbers for the 68 SNPs while HLA_DRB1 indicates all *HLA-DRB1* alleles coded as a biallelic marker: HLA-DRB1*0301/non-HLA-DRB1*0301. The numbers shown below the marker names correspond to the marker numbering in Tables 1, S1, and S3 for ease of interpretation. The grey ticks indicate SNP location to scale. The triangular units designate haplotype blocks. The open rectangles represent the 14 markers that show significant association in the cohort after permutation testing; the green rectangle highlights our class III signal, SNP *rs419788*; and the red rectangle designates our class II signal, *HLA-DRB1*. The 53 markers used for the EHH analyses and haplotype bifurcation plots are designated by open squares. The degree of correlation between pairs of markers is indicated by the correlation coefficient *r* ^2^ (where *r* ^2^ = 0 = no correlation, white; 0 < *r* ^2^ < 1, shades of grey; *r* ^2^ = 1 = complete correlation, black).

Association testing of the MHC region SNPs also identified significant evidence of association to SLE (Table 1 for associated markers and Table S3 for all MHC SNPs). The SNP with the most significant association, *rs419788* (nominal *p* = 4.3 × 10^−8^, permuted *p* < 0.0001) was of similar strength to that of the HLA-DRB1*0301 allele, with odds ratios (ORs) and 95% confidence intervals (CIs) of 2.0 (1.6–2.6) and 2.3 (1.7–3.2), respectively. This SNP is located within intron 6 of the class III gene, superkiller viralicidic activity 2-like (Saccharomyces cerevisiae) (*SKIV2L*), and is located approximately 500 kb telomeric to the *HLA-DRB1* gene. Of the other 12 SNPs that were significantly associated with SLE (nominal *p* = 4.0 × 10^−4^ to 2.5 × 10^−7^; permuted *p* = 0.03 to <0.0001), one was located in the class I region between *HLA-B* and *MICA*, seven were located in the class III region, and four were situated in the class II region (Table 1; Figure S1). Specifically, the seven associated class III SNPs were located in or close to the following genes: the *TNF* promoter, *BAT3*, *SLC44A4*, *EHMT2*, *TNXB*, *GPSM3*, and *NOTCH4*. One of the four class II associated SNPs was close to *HLA-DRA*, two were between *HLA-DRB1* and *HLA-DQA1* and one was in intron 1 of *HLA-DQA1*. The correlation between all 68 SNPs and *HLA-DRB1* in our UK SLE cohort is illustrated in Figure 1. The markers showing significant association are highlighted.

### Conditional Analyses Identify Two Independent Association Signals in the MHC

In order to establish whether the two most associated signals identified in this association-mapping experiment are likely to represent a single causal allele or independent risk factors, we first examined the association data conditioned upon the presence of the HLA-DRB1*0301 allele. We found that four of the 13 associated SNPs showed evidence of signals independent of HLA-DRB1*0301 in our dataset, the strongest of which was *rs419788* (Table 1). We therefore conditioned the three remaining SNPs (*rs2523589*, *rs1052486*, and *rs605203*) on *rs419788* to assess whether these signals are truly independent of each other or show association due to LD with *rs419788*. In addition, we included *HLA-DRB1* in stepwise conditional regression analyses performed on the SNPs showing association independent of *HLA-DRB1* (unpublished data). These analyses demonstrated that the observed association signals at *rs2523589*, *rs1052486*, and *rs605203* were predominantly dependent upon the association at *rs419788*, and suggested that there are two major independent association signals in the MHC in UK SLE: *HLA-DRB1* and *rs419788*. The independence of the association signals at *HLA-DRB1* and *rs419788* is further supported by the observation that there is only modest LD between these two (*r* ^2^ = 0.24). There was no association with any other *HLA-DRB1* allele and the four SNPs independent of HLA-DRB1*0301 (TRANSMIT, unpublished data).

The association of the tumour necrosis factor gene promoter SNP *TNF-308G/A* with SLE is lost after conditioning for HLA-DRB1*0301 in our cohort. If we perform the reverse analysis and condition HLA-DRB1*0301 on the presence of the *TNF* promoter SNP, we find that the association remains, confirming that our *TNF* association is secondary to that of HLA-DRB1*0301.

### Genotypic Risk for Class II and Class III Association Signals

Having established independent association at the allelic level with HLA-DRB1*0301 and *rs419788-T* in our UK SLE cohort, we wanted to further determine the genotypic risk conferred by these variants and hence gain insight into their underlying mode of inheritance in lupus. We used case-control and family-based analyses to assess genotypic risk at *HLA-DRB1*, while the family-based test alone was used for *rs419788*. Common family-based tests of LD, such as those used in this study (Genehunter), measure transmission distortion based on allele counts rather than genotype counts; the former has been shown to be more powerful under additive models, while the latter has greater power under recessive or dominant genetic models [27]. The genotype-pedigree disequilibrium test (geno-PDT) determines LD between a locus genotype and disease by comparing genotypes that are transmitted from parent to proband with those that are not [27]. We used the geno-PDT to assess genotypic risk for our class II and class III association signals: *HLA-DRB1* and the SNP *rs419788*. In the case-control analysis for *HLA-DRB1*, ORs with 95% CI were calculated and Fisher's exact test employed to assess statistically significant differences between *HLA-DRB1* genotypes in lupus probands and healthy controls. For *HLA-DRB1*, the alleles were coded as follows: HLA-DRB1*0301, HLA-DRB1*1501, HLA-DRB1*X where *X* represents all *HLA-DRB1* alleles other than HLA-DRB1*0301, and HLA-DRB1*1501. We included HLA-DRB1*1501 in the analysis, even though we find no allelic association in our cohort, because previous studies have shown a greater risk for lupus in individuals who are compound heterozygotes for HLA-DRB1*0301- and HLA-DRB1*1501-containing haplotypes [25,28]. Overall the results are consistent with a dominant effect from HLA-DRB1*0301 (Table 2) and a dose-dependent (additive) effect from *rs419788-T* (Table 3). Specifically, both case-control and geno-PDT demonstrate that there is no dose-dependent increase in disease risk for HLA-DRB1*0301. Rather, it appears that the presence of a single copy of HLA-DRB1*0301 alone is sufficient to increase susceptibility to disease. Moreover the *0301/X* genotypes constitute the greatest risk in our cohort rather than the *0301/1501* heterozygotes. Genotypes containing HLA-DRB1*1501 in the absence of HLA-DRB1*0301 revealed no significant association in our cohort.

**Table 2:**
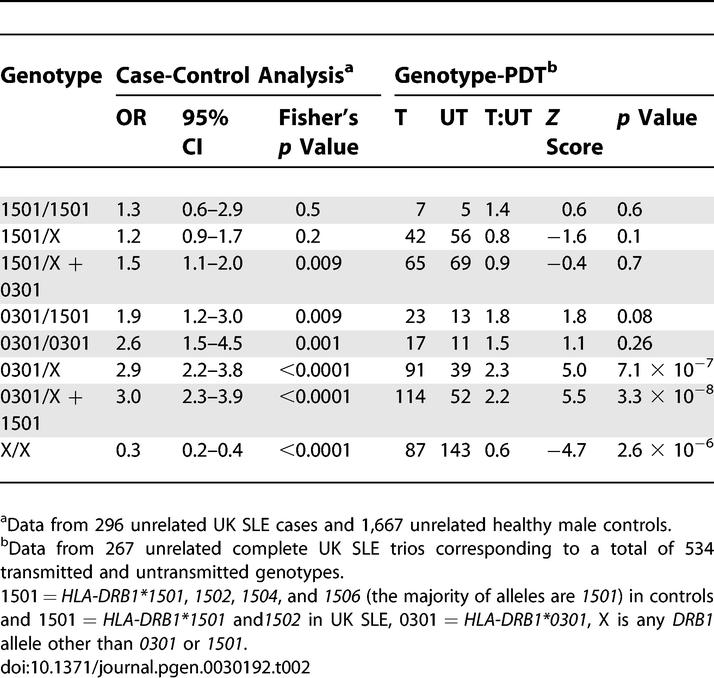
Case-Control and Family-Based Genotypic Risk for *HLA-DRB1* in UK SLE Trios

**Table 3:**
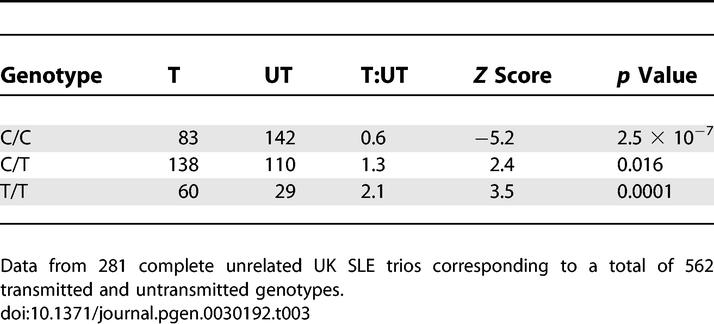
Genotype-Pedigree Disequilibrium Test for SNP *rs419788* in 281 UK SLE Trios

All three *rs419788* genotypes demonstrated significant association in our lupus families (Table 3). The common *CC* genotype was significantly under transmitted, while the rare *T* allele displayed dose-dependent over transmission to lupus probands.

### Characterization of HLA-DRB1*0301 Risk Haplotype

**Figure 2:**
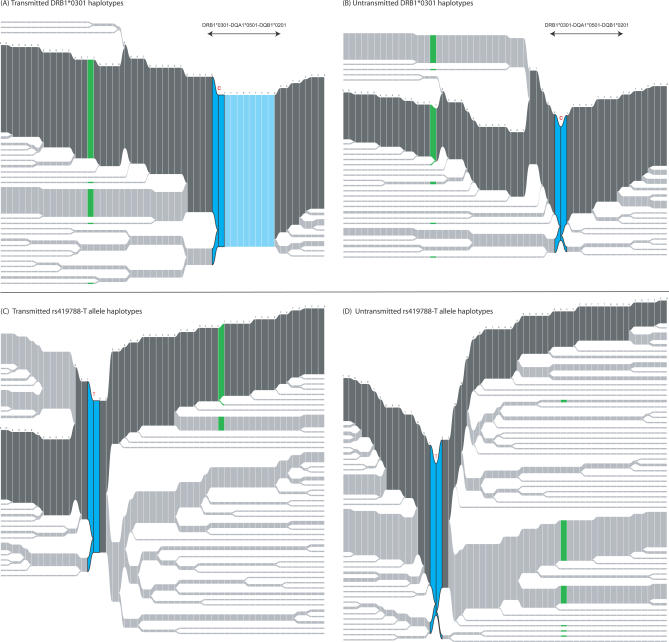
Structure of Transmitted and Untransmitted HLA-DRB1*0301 and *rs419788-T* Allele Haplotypes Haplotype bifurcation plots were constructed using 120 randomly selected parental chromosomes from each of the four datasets for comparative purposes except (B),where there were only 90 chromosomes in the entire dataset: (A) T HLA-DRB1*0301 haplotypes, (B) UT HLA-DRB1*0301 haplotypes, (C) T *rs419788-T* allele haplotypes, and (D) UT *rs419788-T* allele haplotypes. The allelic composition of the most common haplotype in each subset is shown: the core allele is represented as a dark blue double bar indicating haplotypes to the right and to the left of the core; otherwise, the common haplotype is depicted by dark grey bars. In parts (A) and (B), the *rs419788-T* allele in class III that shows association independent of HLA-DRB1*0301 in our cohort is indicated in green, while in parts (C) and (D), the allele HLA-DRB1*0301 is shown in green. The key difference between HLA-DRB1*0301 T and UT haplotypes lies within the class II region of the MHC. All HLA-DRB1*0301 T haplotypes are identical across a 180 kb region defined by eight SNPs (light blue), whereas the corresponding region within UT HLA-DRB1*0301 haplotypes exhibits significant recombination. This conserved class II interval encompasses only three expressed genes: *HLA-DRB1*, *HLA-DQA1*, and *HLA-DQB1*. Given the strong LD exhibited by HLA-DRB1*0301 haplotypes, the allelic composition of this risk region is known to be HLA-DRB1*0301-HLA-DQA1*0501-HLA-DQB1*0201. Both T and UT *rs419788-T* allele haplotypes show similar structure overall. The *rs419788-T* allele is clearly present on HLA-DRB1*0301 and non-HLA-DRB1*0301-containing haplotypes, lending credence to our observation that *rs419788-T* or another variant in LD with it constitutes an association signal independent of HLA-DRB1*0301 in our UK SLE cohort. (A) 120 from a total of 176 parental chromosomes, (B) 90 from a total of 90 parental chromosomes, (C) 120 from a total of 284 parental chromosomes, and (D) 120 from a total of 182 parental chromosomes.

Next, we wanted to further delimit the MHC class II association signal that we have detected at *HLA-DRB1*. We used phased parental genotype data to compare the allelic composition of HLA-DRB1*0301-bearing haplotypes that were transmitted (T) to affected probands to those that were not transmitted (or untransmitted, UT) with the aim of identifying differences that could delineate the lupus susceptibility interval(s) arising from this haplotype (summarized in Figures 2A, 2B, and S2). We observed a striking difference between transmitted and untransmitted chromosomes within the class II region: nearly all transmitted HLA-DRB1*0301 haplotypes (99%) are identical across a 180 kb region defined by eight SNPs, whereas the corresponding region within untransmitted HLA-DRB1*0301 haplotypes exhibits significant recombination. These data strongly suggest the existence of a risk haplotype that, interestingly, contains only three expressed genes: *HLA-DRB1*, *HLA-DQA1*, and *HLA-DQB1*. Furthermore, we can confidently define the allelic composition of this risk haplotype, as these three genes are in strong LD and occur in one common haplotype in Caucasians: HLA-DRB1*0301-HLA-DQA1*0501-HLA-DQB1*0201. Thus, we hypothesize that the specific combination of all three alleles is required to confer disease risk in lupus or that disease susceptibility lies with either HLA-DRB1*0301 or the *HLA-DQ* alleles. We do not have sufficient numbers of recombinant chromosomes in this risk region to further delimit this signal: 2/176 (1.1%) transmitted HLA-DRB1*0301 haplotypes are recombinant at *HLA-DQA1-HLA-DQB1*; 3/178 (1.7%) transmitted haplotypes identical across *HLA-DQA1-HLA-DQB1* do not possess HLA-DRB1*0301.

**Figure 3:**
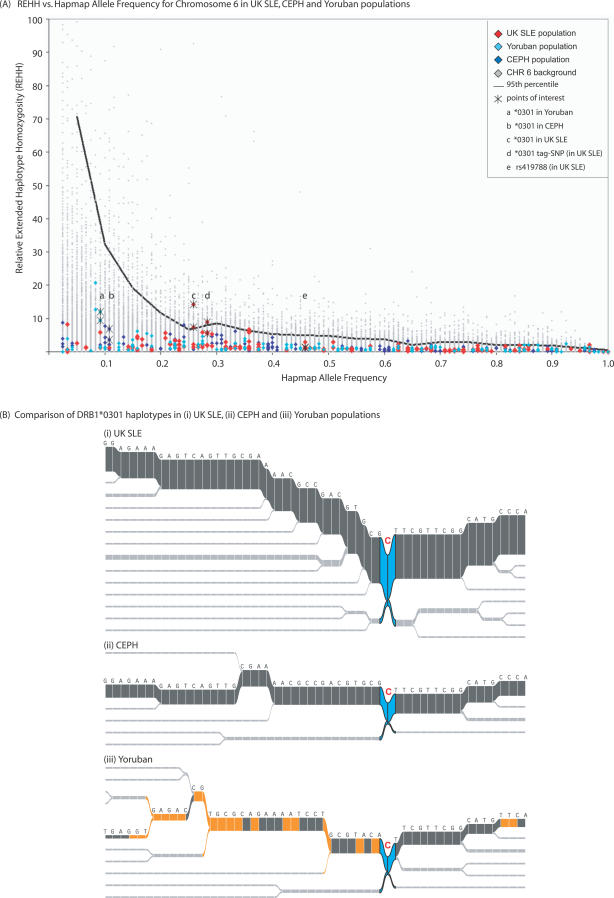
REHH versus Frequency Analysis and Comparison of HLA-DRB1*0301 Haplotype Bifurcation Plots in UK SLE, CEPH, and Yoruba Populations (A) Composite REHH versus frequency analysis comparing CEPH with UK SLE and Yoruba populations generated in SWEEP. REHH is shown on the vertical axis and haplotype frequency on the horizontal axis. Background CEPH genotype data for Chromosome 6 is indicated by grey data points; the 53 SNPs common to the UK SLE, CEPH, and Yoruba datasets are shown as red, blue, and turquoise data points, respectively. The 95th percentile for background variation in CEPH is indicated. The position of the HLA-DRB1*0301 allele is shown in all three cohorts (a, b, and c). The alleles *rs2187668-T* (HLA-DRB1*0301 tag SNP in UK SLE) (d) and HLA-DRB1*0301 in UK SLE are the only core markers observed above the 95th percentile. The associated class III SNP, *rs419788-T*, is also indicated (e). We can only examine evidence for positive selection in CEPH, as these are the data we have used to assess background variation. There is no evidence for positive selection of the HLA-DRB1*0301 allele in CEPH. This allele is enriched in our lupus cohort (21% of parental chromosomes), and displays greater extended homozygosity when compared with HLA-DRB1*0301-bearing haplotypes in CEPH and Yoruba. (B) Comparison of HLA-DRB1*0301 haplotype bifurcation plots for (i) UK SLE, (ii) CEPH, and (iii) Yoruba populations. We show preservation of the common HLA-DRB1*0301 haplotype in CEPH and UK SLE, while that seen in the Yoruba is significantly different (differences indicated in yellow, core allele shown in dark blue). The class II regions of all three populations are essentially identical across our chosen SNPs; the main differences lie in class III. 120 (of 1,256) randomly selected transmitted and untransmitted parental UK SLE chromosomes used for haplotype bifurcation plot for comparison with 120 total CEPH and 120 total Yoruba chromosomes.

The composite relative extended haplotype homozygosity (REHH) versus frequency plot for UK SLE; Utah residents with ancestry from northern and western Europe (CEPH); and Yoruba in Ibadan, Nigeria (Yoruba) populations is shown in Figure 3A. We can only comment on evidence for positive selection in CEPH individuals, as we have used this population alone to assess background variation on Chromosome 6. The SLE and Yoruba cohorts are shown for comparative purposes. We find no evidence of positive selection for HLA-DRB1*0301 in the CEPH population. However, this allele is enriched in our lupus cohort (21% of parental chromosomes) and displays greater extended homozygosity when compared with HLA-DRB1*0301-bearing haplotypes in CEPH and Yoruba. Hence, the HLA-DRB1*0301 allele in lupus is observed as an outlier on the plot when compared to background variation in CEPH. These data support our previous observations (outlined above) of the highly conserved nature of HLA-DRB1*0301 haplotypes in lupus. In addition, the haplotype bifurcation plots centered on HLA-DRB1*0301 for UK SLE, CEPH, and Yoruba populations in Figure 3B illustrate preservation of the common HLA-DRB1*0301 haplotype in CEPH and UK SLE, while that seen in the Yoruba is significantly different. The class II regions of all three populations are essentially identical across our chosen SNPs; the main differences lie in class III. The difference in African populations in the class III region is one possible explanation for the lack of evidence for an association between HLA-DRB1*0301 and SLE in African or African American populations. However, HLA-DRB1*0301 has a lower frequency (∼7%–10%) in African populations compared with Europeans (∼13%), and the number of HLA association studies conducted in African populations is very limited.

### Characterization of Class III Region Risk Haplotype

Our data reveal a second independent signal at the MHC in SLE arising from the T allele of SNP *rs419788* in intron 6 of the class III gene, *SKIV2L*. Further evidence supporting the independence of the *rs419788-T* and HLA-DRB1*0301 alleles is provided by the moderate LD between these two variants (*r* ^2^ = 0.24) coupled with our data demonstrating that only 47% of *rs419788-T* allele-bearing haplotypes contain HLA-DRB1*0301.

**Figure 4:**
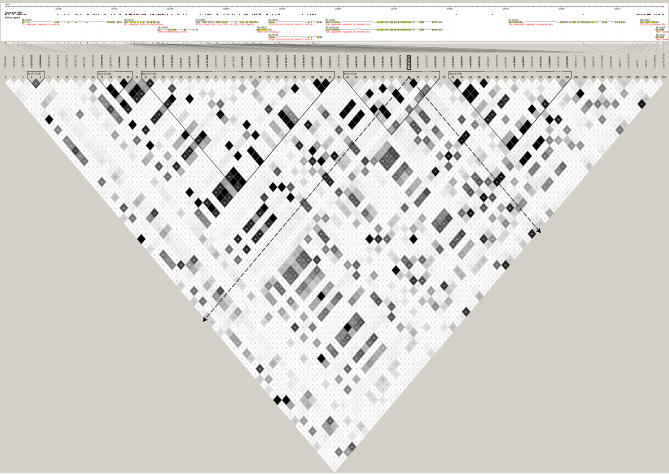
LD Plot of HapMap CEPH Data Centered on the Associated Class III SNP *rs419788* One hundred and twenty kilobases of the MHC class III region centered on *rs419788* (boxed) in HapMap CEPH families is illustrated. The grey ticks indicate SNP location with gene location shown above and rs numbers below. The triangular units designate haplotype blocks. The degree of correlation (LD) between pairs of markers is indicated by the correlation coefficient *r* ^2^ (where *r* ^2^ = 0 = no correlation, white; 0 < *r* ^2^ < 1, shades of grey; *r* ^2^ = 1 = complete correlation, black). The dashed arrows depict LD between *rs419788* and surrounding SNPs. Significant LD (*r* ^2^ > 0.8) is observed with *rs419788* and three further SNPs within *SKIV2L*, two SNPs in *STK19*, and one SNP in *CFB*.

The structure and composition of T and UT haplotypes anchored at *rs419788-T* were essentially identical (Figures 2C, 2D, and S2), and hence not informative in delimiting our class III signal. Therefore, we examined the LD structure around our associated class III SNP to better define our disease risk interval. In our lupus dataset the *rs419788-T* allele resides on three of seven haplotypes present within a large block of six SNPs exhibiting strong LD. This haplotype block encompasses roughly 270 kb containing class III genes from *SLC44A4* to *AGER*, including the *RCCX* module. Next, we analyzed the haplotype block structure of this region in CEPH families using SNP data dumped from the International HapMap Project (http://www.hapmap.org/). The greater density of SNP typing available in the HapMap CEPH population compared to our current UK SLE map allowed us to potentially refine our signal by exploring correlations between our associated SNP and those surrounding it. Analysis of these data (Figure 4) suggests the presence of short-range LD around our associated variant, *rs419788*, in CEPH families, encompassing approximately 40 kb of the genome which includes the five genes: complement factor B (*CFB*), RD RNA binding protein (*RDBP*), *SKIV2L*, dom-3 homolg Z (C. elegans) (*DOM3Z*), and serine/threonine kinase 19 (*STK19*), and does not include the complement *C4* locus. Furthermore, assessment of marker association in our lupus dataset demonstrates that after conditioning for HLA-DRB1*0301, the only markers that retain association signals are telomeric of *SKIV2L*, suggesting that complement *C4*, which is centromeric to this gene, may not be responsible for our independent class III signal.

### Subphenotype Analysis

In order to gain further insight into disease pathogenesis, we examined common lupus subphenotypes. Such subsets are more homogeneous than lupus per se and thus maybe enriched for specific predisposing variants. In addition, one might expect a close association between MHC class II alleles and autoantibody subsets in lupus if these are indeed causal variants, given their role in antigen presentation and subsequent humoral immunity. We therefore tested our two main MHC association signals, HLA-DRB1*0301 and *rs419788*, for association with renal disease and autoantibody subsets in our lupus cohort.

We found that HLA-DRB1*0301 was associated with the presence of anti-Ro and anti-La antibodies in our UK SLE cohort, with the latter showing the greatest evidence of association (anti-La nominal *p* < 0.001 compared with anti-Ro nominal *p* < 0.025). We found no association of HLA-DRB1*0301 with renal disease or any other autoantibody subsets in our dataset (see Table S4 for detailed results).

Genotypes of the SNP *rs419788* were not associated with any of the tested lupus subphenotypes after controlling for the effect of HLA-DRB1*0301 (unpublished data).

## Discussion

We present the first family-based SNP association study of the MHC in SLE. We have genotyped 69 markers (*HLA-DRB1* and 68 SNPs) across 2.4 Mb of the MHC, encompassing class III and class II, in a cohort of 314 UK Caucasian SLE trios. Transmission disequilibrium testing of these data has shown predominant association with the alleles HLA-DRB1*0301 and *rs419788-T*, together with 12 other MHC SNPs. Moreover, using conditional analyses, we have shown that the two primary signals of association at the MHC are independent of each other. Specifically, one signal arises from HLA-DRB1*0301 in class II and the other from the *T* allele of SNP *rs419788* in the class III gene *SKIV2L*.

Examination of bifurcation plots for T and UT HLA-DRB1*0301-containing haplotypes has enabled delineation of our class II association signal to a 180 kb region encompassing HLA-DRB1*0301-HLA-DQA1*0501-HLA-DQB1*0201. These data substantially refine that previously published by Graham et al. in 2002 [25], where the lupus susceptibility interval within HLA-DRB1*0301-containing haplotypes could only be delimited to a 1 Mb region encompassing class II and class III. The precise causal variant(s) within this region remains to be determined, as the three implicated alleles exhibit strong LD with few recombination events separating them (two out of 176 transmitted HLA-DRB1*0301 chromosomes in our dataset). However, all three allelic variants represent attractive functional candidates in lupus susceptibility for their role in antigen presentation and stimulation of the adaptive immune response.

Our association of HLA-DRB1*0301 with lupus concurs with published data in Caucasian cohorts and is well established [16]. While our lack of association with HLA-DRB1*1501 and HLA-DRB1*0801 is consistent with previous data from the UK [29], Spain [30], the Netherlands [31], Sweden [32], Mexico [33], and the US [34], it conflicts with that of other US groups [25,35]. Interestingly, we demonstrate a trend, though not statistically significant, for undertransmission of HLA-DRB1*0701—a result also observed in prior UK and Canadian lupus studies [29,36]. Moreover, a negative association of HLA-DRB1*0701 has been reported in other autoimmune diseases including Graves disease [37,38], type 1 diabetes [39], and rheumatoid arthritis [40].

It appears that the conflicting results between UK SLE and previous US (Minnesota [MN]) [25] SLE data stem from differences in *HLA-DRB1* allele frequency in the probands of each cohort. The reason for this is unclear. A comparison between UK and MN SLE cohorts (Table 4) reveals that UK SLE cases are enriched for HLA-DRB1*0301 but not HLA-DRB1*0801 or HLA-DRB1*1501 when compared to a UK control population. In contrast, MN SLE cases are enriched for HLA-DRB1*0301-DQB1*0201, DRB1*0801-DQB1*0402, and DRB1*1501-DQB1*0602 inferred haplotypes when compared to MN controls [25]. There is no statistically significant difference in the aforementioned HLA class II alleles/haplotypes between UK and MN control populations that could account for the disparity seen in the respective lupus cohorts. Differences in disease severity and subphenotype frequency between the two populations could account for the observed discrepancy. From the limited data available we found that the presence of renal disease appears to be similar in both cohorts (UK SLE 36% compared with MN SLE 40%), while the gender ratios are significantly different (female: male UK SLE 11:1 compared with MN SLE 57:1, Chi square *p* value < 0.001). We were unable to compare other lupus subphenotypes. Furthermore, closer inspection of these data reveals that microsatellite-inference of *HLA-DRB1* alleles in the MN SLE dataset may underestimate the frequency of HLA-DRB1*0301 and overestimate that of HLA-DRB1*1501, thus diminishing the effect of the former and enhancing that of the latter. It is also possible that the MN SLE cohort shows greater racial heterogeneity in comparison to our UK SLE cohort, despite both being characterized as Caucasian.

**Table 4:**
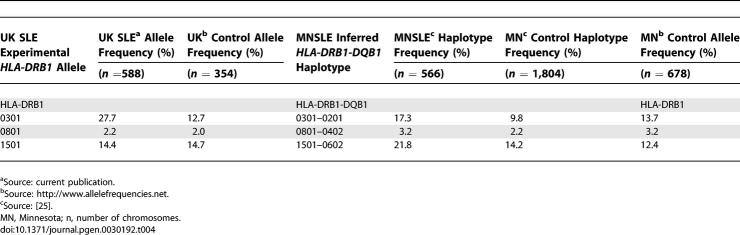
Comparison of *HLA-DRB1* Allele/Haplotype Frequencies in UK SLE and US (Minnesota) SLE Cohorts

Previous studies have demonstrated increased risk for lupus in individuals carrying particular combinations of microsatellite-inferred *HLA-DRB1-HLA-DQB1* haplotypes [25,28]. The highest risk genotype was found to be the compound heterozygote HLA-DRB1*0301-DQB1*0201/HLA-DRB1*1501-DQB1*0602, while HLA-DRB1*0301-DQB1*0201-containing genotypes demonstrated a dose-dependent effect in increasing lupus susceptibility [25,28]. In the present study, we have examined genotypic risk at the classically typed *HLA-DRB1* locus and in contrast to the aforementioned data of Graham et al. [25,28] we have shown a likely dominant effect of the associated allele, HLA-DRB1*0301. The case-control and family-based analyses for *HLA-DRB1* also show the greater power of the former to detect significant association (Table 2). Specifically, all genotypes containing HLA-DRB1*0301 show increased transmission to lupus probands; however, homozygotes show no greater risk compared with heterozygotes, as would be expected under additive or multiplicative models. Thus, a dominant model of inheritance, requiring the presence of a single copy of the disease-predisposing variant alone, likely underlies the susceptibility conferred by HLA-DRB1*0301 in UK SLE. Such a model would fit an antigen presentation hypothesis where susceptible individuals carrying an HLA-DRB1*0301 allele are able to present auto-antigens to CD4+ lymphocytes, thus stimulating an autoimmune response. The differences between our UK SLE and the previously published US SLE data may reflect disease, ethnic, and haplotypic heterogeneity.

Interestingly, analysis of genotypic risk at the associated class III marker, *rs419788*, suggests an additive (dose-dependent) pattern of inheritance for the rare *T* allele, where one copy confers a low risk of disease and two copies results in greater susceptibility. The different inheritance patterns for our class II and class III association signals provide further evidence for their independence.

A variety of *HLA-DR* and *HLA-DQ* alleles have been associated with autoantibody subsets in ethnically diverse populations of lupus. The strongest associations have been demonstrated between anti-Ro and anti-La antibodies and *HLA-DR3* and *HLA-DQ2* (HLA-DQB1*0201), which are in strong LD [41–45] in case-control studies. Here, we confirm the association of HLA-DRB1*0301 with anti-Ro and anti-La antibody production in our family-based cohort.

Examination of LD structure around our second independent association, *rs419788-T* in class III, coupled with the results of our conditional analysis, suggests that this signal could also be delimited to a relatively narrow genomic interval of about 40 kb given further SNP mapping in our cohort. This region includes the genes *CFB*, *RDBP*, *SKIV2L*, *DOM3Z*, and *STK19*, but does not include complement *C4*. Thus, complement *C4* null alleles, which have been implicated in lupus pathogenesis, may not be responsible for our class III signal. We conclude, therefore, that our family-based mapping study has potentially revealed a hitherto unknown lupus susceptibility interval in the class III region of the MHC. However, we cannot conclusively exclude association at complement *C4*/*RCCX* without direct determination of *C4* polymorphism/copy number in our cohort.

With respect to the genes implicated in our study, CFB is a vital component of the alternate complement pathway and disregulation may clearly affect the inflammatory response [46]. RD and Skiv2l are proteins potentially involved in RNA processing. The RD protein forms part of a negative elongation factor (NELF) complex that represses RNA polymerase II transcript elongation, while Skiv2l is a DEAD box protein with possible function as an RNA helicase. The function of Dom3z is currently unknown, although the homologous yeast protein binds nuclear exoribonuclease. Moreover, its ubiquitous expression suggests a housekeeping role. STK19 is a protein kinase of unknown function with primary nuclear localization [47]. Interestingly, *RDBP* and *SKIV2L* are found to be highly expressed in T lymphocytes, B lymphocytes, and dendritic cells (SymAtlas, http://symatlas.gnf.org/SymAtlas/).

A number of studies have demonstrated conflicting evidence for and against association with various *TNF* locus polymorphisms in SLE [48]. A recent meta-analysis of the *TNF-308G/A* promoter polymorphism in SLE [48] revealed evidence of association for the minor allele (A) in European populations; however, this study did not account for LD with class II alleles. On conditioning our dataset for HLA-DRB1*0301, we find that the *TNF* promoter signal is lost, suggesting that this association is not independent and is due to LD with HLA-DRB1*0301 (or another variant in LD with HLA-DRB1*0301).

In summary, we have found association with two distinct and independent variants within the class II (HLA-DRB1*0301) and class III (*SKIV2L*) regions of the MHC in UK SLE trios. We can delimit our class II signal in lupus to three genetic variants (HLA-DRB1*0301-HLA-DQA1*0501-HLA-DQB1*0201) that may confer disease risk in combination or as separate signals. Our class III signal importantly excludes independent association at the *TNF* promoter polymorphism *TNF-308G/A* and potentially provides a novel locus for further study.

## Materials and Methods

### Study cohorts.

*SLE families*. The cohort comprises 314 complete SLE trios (that is, mother, father, and affected lupus proband) collected as previously described [49]. All study participants are European Caucasian on the basis of grandparental origin. All 314 lupus probands (288 female, 26 male) fulfill the revised American College of Rheumatology (ACR) criteria for SLE [50], 36% of whom have a diagnosis of lupus nephritis. Written consent was obtained from all study participants and ethical approval for this study was obtained from the Multi-Centre Research Ethics Committee (MREC 2 June 1998).

### Healthy controls.

The control population for the *HLA-DRB1* genotypic risk case-control analysis constitutes 1,667 healthy males of Northern European origin. The individuals are potential hematopoietic stem cell donors and were typed to four digits for *HLA-DRB1* at the Anthony Nolan Trust, UK for this purpose. The level of resolution used for the typing of HLA-DRB1*15 alleles in these healthy controls resulted in the ambiguous allele string HLA-DRB1*1501/*1502*/*1504*/*1506*. However, it is likely that the great majority are HLA-DRB1*1501. There is no gender bias in *HLA-DRB1* allele frequencies, so although we have used a male control cohort, the frequencies would be expected to be the same in a similar female cohort (Steven Marsh, personal communication).

### SNP genotyping.

Eighty-six SNPs were chosen for genotyping in our mapping study. Specifically, we selected 40 MHC class II and class III haplotype tagging SNPs from a preliminary MHC SNP map [51] that had previously shown robust genotyping efficacy. In addition, we typed 36 MHC class II tag SNPs from a subsequent high-resolution MHC study [52]. We also included the *TNF-308G/A* promoter SNP, together with nine further SNPs in the region of *HLA-B* and *MICA* obtained from the database, dbSNP (http://www.ncbi.nlm.nih.gov/projects/SNP/). The latter SNPs had not been well characterized. All variants were typed in the entire cohort (*n* = 942). The SNPs span approximately 2.4 Mb of the MHC from the class I gene, *KIAA1949* to the class II pseudogene, *HLA-DPB2* and thus encompass MHC class III and class II. SNP genotyping was performed at the Broad Institute of MIT and Harvard and at Imperial College London by matrix-assisted laser desorption/ionisation time-of-flight (MALDI-TOF) mass spectrometry using the Sequenom MassARRAY platform as previously described [53]. SNPs that failed Sequenom typing were retyped by KBiosciences (http://www.kbioscience.co.uk/) using their in-house SNP genotyping methodology, KASPar (http://www.kbioscience.co.uk/genotyping/index.htm), a competitive allele-specific PCR technique.

### HLA-DRB1 genotyping.

*HLA-DRB1* typing was performed at the Anthony Nolan Trust, UK. All samples (*n* = 942 UK SLE trios and *n* = 1,667 controls) were genotyped using LABType SSO (sequence-specific oligonucleotide) typing technology according to the manufacturer's written recommendations (http://www.onelambda.com). Briefly, a locus-specific biotinylated PCR amplicon is produced, denatured, and rehybridized to complementary oligonucleotide probes conjugated to fluorescently coded beads. The bound biotinylated PCR product can be detected using R-phycoerythrin-conjugated streptavidin. A flow analyzer identifies the fluorescent intensity of phycoerythrin on each bead. The assignment of HLA type is based on the reaction pattern compared to patterns associated with known sequences.

High resolution testing was performed using the Dynal AllSet^+^ SSP (sequence-specific primers) DRB1 assay according to the manufacturer's protocol (Invitrogen) for SLE families only. The presence or absence of PCR amplification was detected in a gel electrophoresis step using visualization by ethidium bromide incorporation. Genotypes were determined using SSPTool software.

Samples that could not be resolved to four digits using SSO and PCR-SSP were analyzed by DNA sequencing of exon 2 of *HLA-DRB1*. Primers, reagents, and protocols were provided by The Anthony Nolan Trust, UK (primer sequences are available on request). Specific *HLA-DRB1* alleles were assigned by comparing the resultant sequence with reference sequence from the IMGT/HLA Database [54].

### Data analysis.

Mendelian inconsistencies were removed using PedCheck [55]. Families in which more than eight markers demonstrated Mendel errors were removed from further analysis. Markers with less than 80% genotyping efficiency and markers where more than eight families showed Mendel errors were also excluded from analysis. Two markers located within the SLE associated class II region (*rs2239802* in intron 4 of *HLA-DRA* and *rs6457594* in the region between *HLA-DRB9* and *HLA-DRB5*) show deviation from Hardy–Weinberg equilibrium (HWE), which may reflect an undetected SLE association or systematic genotyping error. HWE was assessed in parental samples in our cohort. There is currently no uniform opinion in the community regarding the inclusion or exclusion of SNPs that show deviation from HWE, hence we elected to include these markers in the final analysis.

Sixty-eight out of the total 86 SNPs passed our quality-control measures (see Table S1 for details). In summary, one SNP was monomorphic in our dataset, four SNPs yielded low genotyping efficiency, and 13 SNPs were excluded for unacceptable Mendel error rate. The mean call rate for all markers post-quality control was 94% (range 83% to 99%).

Family-based association testing was performed using Genehunter TDT (version 2.1) [56] and TRANSMIT (version 2.5) [57]. Haplotypes were constructed and permutation testing performed using Haploview (version 3.32) [58]. Significance of association signals in all analyses was based on permutation testing (10,000 permutations). The data are represented both as nominal and permuted *p* values. ORs with 95% CI for family-based analyses were calculated in PLINK (http://pngu.mgh.harvard.edu/purcell/plink/) [59]. Conditional regression analyses were undertaken using WHAP [60].

The geno-PDT was performed using PDT version 5.1 with default settings [27]. The *HLA-DRB1* alleles were coded into three groups for the geno-PDT and the case-control analysis: HLA-DRB1*0301, HLA-DRB1*1501 and *HLA-DRX* where *HLA-DRX* includes all *HLA-DRB1* alleles other than HLA-DRB1*0301 or HLA-DRB1*1501. The HLA-DRB1*1501 code in the healthy controls represents the allele string HLA-DRB1*1501/*1502*/*1504*/*1506*, as described previously. The HLA-DRB1*1501 code in the lupus probands represents the alleles HLA-DRB1*1501 (84 out of 91 *1501 and *1502 alleles) and HLA-DRB1*1502 (7/91 *1501 and *1502 alleles), as *1504 and *1506 were not present in this population. Fisher's exact test was used to assess significance of association in the case-control analysis.

### Subphenotype analysis.

We looked for association of the HLA-DRB1*0301 allele with autoantibody subsets and renal disease in our cohort using the Chi-square test. We compared cases with and without the subphenotype of interest with DRB1*0301 homozygosity, heterozygosity, combined homozygosity and heterozygosity, and non-DRB1*0301 status. We performed the same analyses for homozygous and heterozygous genotypes of the associated SNP *rs419788*. The autoantibody subsets compared were anti-C1q, IgG, and IgM anti-cardiolipin antibodies (ACLG and ACLM), anti-Ro, anti-La, anti-RNP, anti-Sm, and anti-dsDNA.

### Delineation of associated MHC haplotypes and evidence for positive selection.

We looked for positively selected alleles in our dataset using the long-range haplotype test as measured by extended haplotype homozygosity (EHH), previously described by Sabeti et al. [26]. Essentially, such an analysis allows assessment of positive selection by mining datasets for high frequency extended haplotypes in comparison to the other core haplotypes at a locus.

EHH is defined as the probability that two randomly chosen chromosomes carrying the core haplotype of interest will be identical by descent (homozygosity at all SNPs) for the entire interval from the core to a distance *x*. The REHH is the ratio of the EHH on the tested core haplotype compared with the combined EHH of all the other core haplotypes at the region excluding the tested core; as such, REHH accounts for local variation in recombination rate while EHH does not [26].

The program emphase was employed to assign the phase of parental genotype data and reconstruct missing information. Emphase is a simple phaser similar to the phaser of Excoffier and Slatkin [61]. It is very fast, especially on large datasets, and sufficiently accurate for most genetic applications. EHH analysis was performed on the phased parental data using the software program SWEEP (http://www.broad.mit.edu/mpg/sweep/index.html).

### Haplotype bifurcation plots.

We represent the breakdown of LD on core haplotypes using haplotype bifurcation diagrams generated in the program TREE [62] (also explained in [52]).

### REHH versus frequency plots.

Fifty-three SNPs (identified in Figure 1) are common to our dataset and the CEPH and Yoruba HapMap [63] populations. These three datasets, together with CEPH SNP data for Chromosome 6 in its entirety, were used to generate separate REHH versus frequency plots in SWEEP. The plots from the four cohorts were combined for visual, not statistical, comparison. Evidence for positive selection was quantitatively assessed in CEPH individuals, as this population alone was used to assess background variation on Chromosome 6. The UK SLE and Yoruba cohort data are shown for comparison. The 95th percentile based on total CEPH Chromosome 6 SNP data is shown.

## Supporting Information

Figure S1 OR Plot for Associated MHC Markers in 314 UK SLE Trios OR with 95% CI and marker name in genomic order (class I to class II, left to right) are indicated on the vertical and horizontal axes respectively. The greatest ORs are seen in the class III and class II regions.
(42 KB PDF)

Figure S2 Structure of *All* Transmitted and Untransmitted HLA-DRB1*0301 and *rs419788-T* Allele Haplotypes Haplotype bifurcation plots constructed for all (A) transmitted (T) HLA-DRB1*0301 haplotypes, (B) untransmitted (UT) HLA-DRB1*0301 haplotypes, (C) transmitted *rs419788-T* allele haplotypes, and (D) untransmitted *rs419788-T* allele haplotypes. The allelic composition of the most common haplotype in each subset is shown: the core allele is represented as a dark blue double bar indicating haplotypes to the right and to the left of the core; otherwise, the common haplotype is depicted by dark grey bars. In parts (A) and (B), the *rs419788-T* allele in class III, which shows association independent of HLA-DRB1*0301 in our cohort, is indicated in green, while in parts (C) and (D), the allele HLA-DRB1*0301 is shown in green. The key difference between HLA-DRB1*0301 T and UT haplotypes lies within the class II region of the MHC. All HLA-DRB1*0301 T haplotypes are identical across a 180 kb region defined by eight SNPs (light blue), whereas the corresponding region within UT HLA-DRB1*0301 haplotypes exhibits significant recombination. This conserved class II interval encompasses only three expressed genes: *HLA-DRB1*, *HLA-DQA1*, and *HLA-DQB1*. Given the strong LD exhibited by HLA-DRB1*0301 haplotypes, the allelic composition of this risk region is known to be HLA-DRB1*0301-HLA-DQA1*0501-HLA-DQB1*0201. Both T and UT *rs419788-T* allele haplotypes show similar structure overall. The *rs419788-T* allele is clearly present on HLA-DRB1*0301 and non-HLA-DRB1*0301-containing haplotypes, lending credence to our observation that *rs419788-T* or another variant in LD with it constitutes an association signal independent of HLA-DRB1*0301 in our UK SLE cohort.
(A) total of 176 parental chromosomes, (B) total of 90 parental chromosomes, (C) total of 284 parental chromosomes, and (D) total of 182 parental chromosomes.
(338 KB PDF)

Table S1 Quality Control Information for Four-Digit *HLA-DRB1* Typing and All 86 SNPs Chosen for Genotyping in a Cohort of 314 UK SLE Trios (26 KB XLS)

Table S2 OR and TDT Data for Four-Digit *HLA-DRB1* Typing in a Cohort of 314 UK SLE Trios (20 KB XLS)

Table S3 Complete Single Marker (SNP) and HLA-DRB1*0301 Association Data in a Cohort of 314 UK SLE Trios (34 KB XLS)

Table S4 Association of HLA-DRB1*0301 Genotypes with SLE Subphenotypes in 314 UK SLE Probands (32 KB DOC)

### Accession Numbers

The Online Mendelian Inheritance in Man (OMIM, http://www.ncbi.nlm.nih.gov/sites/entrez?db=omim) accession numbers for the genes described in this study are as follows: AGER, 600214; BAT3, 142590; C4A, 120810; C4B, 120820; CFB, 138470; DOM3Z, 605996; DOM3Z, 605996; EHMT2, 604599; HLA-B, 142830; HLA-DPB2, 120290; HLA-DQA1, 146880; HLA-DQB1, 604305; HLA-DRA, 142860; HLA-DRB1, 142857; KIAA1949, 610990; MICA, 600169; NOTCH4, 164951; RDBP, 154040; RDBP, 154040; SKIV2L, 600478; SLC44A4, 606107; STK19, 604977; STK19, 604977; TNF, 191160; and TNXB, 600985.

## Abbreviations

CEPH: Utah residents with ancestry from northern and western Europe
CI: confidence interval
EHH: extended haplotype homozygosity
geno-PDT: genotype-pedigree disequilibrium test
HLA: human leucocyte antigen
HWE: Hardy–Weinberg equilibrium
LD: linkage disequilibrium
MHC: major histocompatibility complex
OR: odds ratio
REHH: relative extended haplotype homozygosity
SLE: systemic lupus erythematosus
T: transmitted
UT: untransmitted
Yoruba: Yoruba in Ibadan, Nigeria

## References

[pgen-0030192-b001] Shiina T, Inoko H, Kulski JK (2004). An update of the HLA genomic region, locus information and disease associations: 2004. Tissue Antigens.

[pgen-0030192-b002] Horton R, Wilming L, Rand V, Lovering RC, Bruford EA (2004). Gene map of the extended human MHC. Nat Rev Genet.

[pgen-0030192-b003] The MHC sequencing consortium (1999). Complete sequence and gene map of a human major histocompatibility complex. The MHC sequencing consortium. Nature.

[pgen-0030192-b004] Awdeh ZL, Raum D, Yunis EJ, Alper CA (1983). Extended HLA/complement allele haplotypes: evidence for T/t-like complex in man. Proc Natl Acad Sci U S A.

[pgen-0030192-b005] Degli-Esposti MA, Leaver AL, Christiansen FT, Witt CS, Abraham LJ (1992). Ancestral haplotypes: conserved population MHC haplotypes. Hum Immunol.

[pgen-0030192-b006] Aly TA, Eller E, Ide A, Gowan K, Babu SR (2006). Multi-SNP analysis of MHC region: remarkable conservation of HLA-A1-B8-DR3 haplotype. Diabetes.

[pgen-0030192-b007] Wakeland EK, Liu K, Graham RR, Behrens TW (2001). Delineating the genetic basis of systemic lupus erythematosus. Immunity.

[pgen-0030192-b008] Deapen D, Escalante A, Weinrib L, Horwitz D, Bachman B (1992). A revised estimate of twin concordance in systemic lupus erythematosus. Arthritis Rheum.

[pgen-0030192-b009] Vyse TJ, Todd JA (1996). Genetic analysis of autoimmune disease. Cell.

[pgen-0030192-b010] Jacob CO, McDevitt HO (1988). Tumour necrosis factor-alpha in murine autoimmune “lupus” nephritis. Nature.

[pgen-0030192-b011] Charles PJ, Smeenk RJ, De Jong J, Feldmann M, Maini RN (2000). Assessment of antibodies to double-stranded DNA induced in rheumatoid arthritis patients following treatment with infliximab, a monoclonal antibody to tumor necrosis factor alpha: findings in open-label and randomized placebo-controlled trials. Arthritis Rheum.

[pgen-0030192-b012] Shakoor N, Michalska M, Harris CA, Block JA (2002). Drug-induced systemic lupus erythematosus associated with etanercept therapy. Lancet.

[pgen-0030192-b013] Vermeire S, Noman M, Van Assche G, Baert F, Van Steen K (2003). Autoimmunity associated with anti-tumor necrosis factor alpha treatment in Crohn's disease: a prospective cohort study. Gastroenterology.

[pgen-0030192-b014] Forabosco P, Gorman JD, Cleveland C, Kelly JA, Fisher SA (2006). Meta-analysis of genome-wide linkage studies of systemic lupus erythematosus. Genes Immun.

[pgen-0030192-b015] Wong M, Tsao BP (2006). Current topics in human SLE genetics. Springer Semin Immunopathol.

[pgen-0030192-b016] Tsao BP (2004). Update on human systemic lupus erythematosus genetics. Curr Opin Rheumatol.

[pgen-0030192-b017] Vyse TJ, Kotzin BL (1998). Genetic susceptibility to systemic lupus erythematosus. Annu Rev Immunol.

[pgen-0030192-b018] Grumet FC, Coukell A, Bodmer JG, Bodmer WF, McDevitt HO (1971). Histocompatibility (HL-A) antigens associated with systemic lupus erythematosus. A possible genetic predisposition to disease. N Engl J Med.

[pgen-0030192-b019] Waters H, Konrad P, Walford RL (1971). The distribution of HL-A histocompatibility factors and genes in patients with systemic lupus erythematosus. Tissue Antigens.

[pgen-0030192-b020] Pickering MC, Perraudeau M, Walport MJ, Lechler R, Warrens A (2000). HLA and systemic vasculitides, systemic lupus erythematosus and Sjogren's syndrome. HLA in Health and Disease. Second ed.

[pgen-0030192-b021] Batchelor JR, Fielder AH, Walport MJ, David J, Lord DK (1987). Family study of the major histocompatibility complex in HLA DR3 negative patients with systemic lupus erythematosus. Clin Exp Immunol.

[pgen-0030192-b022] Hartung K, Baur MP, Coldewey R, Fricke M, Kalden JR (1992). Major histocompatibility complex haplotypes and complement C4 alleles in systemic lupus erythematosus. Results of a multicenter study. J Clin Invest.

[pgen-0030192-b023] Ayed K, Gorgi Y, Ayed-Jendoubi S, Bardi R (2004). The involvement of HLA -DRB1*, DQA1*, DQB1* and complement C4A loci in diagnosing systemic lupus erythematosus among Tunisians. Ann Saudi Med.

[pgen-0030192-b024] Yang Y, Chung EK, Wu YL, Savelli SL, Nagaraja HN (2007). Gene copy-number variation and associated polymorphisms of complement component C4 in human systemic lupus erythematosus (SLE): low copy number is a risk factor for and high copy number is a protective factor against SLE susceptibility in European Americans. Am J Hum Genet.

[pgen-0030192-b025] Graham RR, Ortmann WA, Langefeld CD, Jawaheer D, Selby SA (2002). Visualizing human leukocyte antigen class II risk haplotypes in human systemic lupus erythematosus. Am J Hum Genet.

[pgen-0030192-b026] Sabeti PC, Reich DE, Higgins JM, Levine HZ, Richter DJ (2002). Detecting recent positive selection in the human genome from haplotype structure. Nature.

[pgen-0030192-b027] Martin ER, Bass MP, Gilbert JR, Pericak-Vance MA, Hauser ER (2003). Genotype-based association test for general pedigrees: the genotype-PDT. Genet Epidemiol.

[pgen-0030192-b028] Graham RR, Ortmann W, Rodine P, Espe K, Langefeld C (2007). Specific combinations of HLA-DR2 and DR3 class II haplotypes contribute graded risk for disease susceptibility and autoantibodies in human SLE. Eur J Hum Genet.

[pgen-0030192-b029] McHugh NJ, Owen P, Cox B, Dunphy J, Welsh K (2006). MHC class II, tumour necrosis factor alpha, and lymphotoxin alpha gene haplotype associations with serological subsets of systemic lupus erythematosus. Ann Rheum Dis.

[pgen-0030192-b030] Sanchez E, Torres B, Vilches JR, Lopez-Nevot MA, Ortego-Centeno N (2006). No primary association of MICA polymorphism with systemic lupus erythematosus. Rheumatology (Oxford).

[pgen-0030192-b031] Rood MJ, van Krugten MV, Zanelli E, van der Linden MW, Keijsers V (2000). TNF-308A and HLA-DR3 alleles contribute independently to susceptibility to systemic lupus erythematosus. Arthritis Rheum.

[pgen-0030192-b032] Jonsen A, Bengtsson AA, Sturfelt G, Truedsson L (2004). Analysis of HLA DR, HLA DQ, C4A, FcgammaRIIa, FcgammaRIIIa, MBL, and IL-1Ra allelic variants in Caucasian systemic lupus erythematosus patients suggests an effect of the combined FcgammaRIIa R/R and IL-1Ra 2/2 genotypes on disease susceptibility. Arthritis Res Ther.

[pgen-0030192-b033] Bekker-Mendez C, Yamamoto-Furusho JK, Vargas-Alarcon G, Ize-Ludlow D, Alcocer-Varela J (1998). Haplotype distribution of class II MHC genes in Mexican patients with systemic lupus erythematosus. Scand J Rheumatol.

[pgen-0030192-b034] Uribe AG, McGwin G, Reveille JD, Alarcon GS (2004). What have we learned from a 10-year experience with the LUMINA (Lupus in Minorities; Nature vs. nurture) cohort? Where are we heading?. Autoimmun Rev.

[pgen-0030192-b035] Tsuchiya N, Kawasaki A, Tsao BP, Komata T, Grossman JM (2001). Analysis of the association of HLA-DRB1, TNFalpha promoter and TNFR2 (TNFRSF1B) polymorphisms with SLE using transmission disequilibrium test. Genes Immun.

[pgen-0030192-b036] Gladman DD, Urowitz MB, Darlington GA (1999). Disease expression and class II HLA antigens in systemic lupus erythematosus. Lupus.

[pgen-0030192-b037] Chen QY, Huang W, She JX, Baxter F, Volpe R (1999). HLA-DRB1*08, DRB1*03/DRB3*0101, and DRB3*0202 are susceptibility genes for Graves' disease in North American Caucasians, whereas DRB1*07 is protective. J Clin Endocrinol Metab.

[pgen-0030192-b038] Simmonds MJ, Howson JM, Heward JM, Cordell HJ, Foxall H (2005). Regression mapping of association between the human leukocyte antigen region and Graves disease. Am J Hum Genet.

[pgen-0030192-b039] Cavan DA, Jacobs KH, Penny MA, Kelly MA, Mijovic C (1993). Both DQA1 and DQB1 genes are implicated in HLA-associated protection from type 1 (insulin-dependent) diabetes mellitus in a British Caucasian population. Diabetologia.

[pgen-0030192-b040] Weyand CM, McCarthy TG, Goronzy JJ (1995). Correlation between disease phenotype and genetic heterogeneity in rheumatoid arthritis. J Clin Invest.

[pgen-0030192-b041] Schur PH (1995). Genetics of systemic lupus erythematosus. Lupus.

[pgen-0030192-b042] Logar D, Vidan-Jeras B, Dolzan V, Bozic B, Kveder T (2002). The contribution of HLA-DQB1 coding and QBP promoter alleles to anti-Ro alone autoantibody response in systemic lupus erythematosus. Rheumatology (Oxford).

[pgen-0030192-b043] Azizah MR, Ainoi SS, Kuak SH, Kong NC, Normaznah Y (2001). The association of the HLA class II antigens with clinical and autoantibody expression in Malaysian Chinese patients with systemic lupus erythematosus. Asian Pac J Allergy Immunol.

[pgen-0030192-b044] Miyagawa S, Shinohara K, Nakajima M, Kidoguchi K, Fujita T (1998). Polymorphisms of HLA class II genes and autoimmune responses to Ro/SS-A-La/SS-B among Japanese subjects. Arthritis Rheum.

[pgen-0030192-b045] Galeazzi M, Sebastiani GD, Morozzi G, Carcassi C, Ferrara GB (2002). HLA class II DNA typing in a large series of European patients with systemic lupus erythematosus: correlations with clinical and autoantibody subsets. Medicine (Baltimore).

[pgen-0030192-b046] Taylor PR, Nash JT, Theodoridis E, Bygrave AE, Walport MJ (1998). A targeted disruption of the murine complement factor B gene resulting in loss of expression of three genes in close proximity, factor B, C2, and D17H6S45. J Biol Chem.

[pgen-0030192-b047] Lehner B, Semple JI, Brown SE, Counsell D, Campbell RD (2004). Analysis of a high-throughput yeast two-hybrid system and its use to predict the function of intracellular proteins encoded within the human MHC class III region. Genomics.

[pgen-0030192-b048] Lee YH, Harley JB, Nath SK (2006). Meta-analysis of TNF-alpha promoter −308 A/G polymorphism and SLE susceptibility. Eur J Hum Genet.

[pgen-0030192-b049] Russell AI, Cunninghame Graham DS, Shepherd C, Roberton CA, Whittaker J (2004). Polymorphism at the C-reactive protein locus influences gene expression and predisposes to systemic lupus erythematosus. Hum Mol Genet.

[pgen-0030192-b050] Tan EM, Cohen A.S, Fries J.F, Masi A.T, McShane D.J, Rothfield N.F, Schaller J.G, Talal N, Winchester R.J (1982). The 1982 revised criteria for the classification of systemic lupus erythematosus. Arthritis Rheum.

[pgen-0030192-b051] Walsh EC, Mather KA, Schaffner SF, Farwell L, Daly MJ (2003). An integrated haplotype map of the human major histocompatibility complex. Am J Hum Genet.

[pgen-0030192-b052] de Bakker PI, McVean G, Sabeti PC, Miretti MM, Green T (2006). A high-resolution HLA and SNP haplotype map for disease association studies in the extended human MHC. Nat Genet.

[pgen-0030192-b053] Jurinke C, van den Boom D, Cantor CR, Koster H (2002). The use of MassARRAY technology for high throughput genotyping. Adv Biochem Eng Biotechnol.

[pgen-0030192-b054] Robinson J, Malik A, Parham P, Bodmer JG, Marsh SG (2000). IMGT/HLA database–a sequence database for the human major histocompatibility complex. Tissue Antigens.

[pgen-0030192-b055] O'Connell JR, Weeks DE (1998). PedCheck: a program for identification of genotype incompatibilities in linkage analysis. Am J Hum Genet.

[pgen-0030192-b056] Spielman RS, Ewens WJ (1996). The TDT and other family-based tests for linkage disequilibrium and association. Am J Hum Genet.

[pgen-0030192-b057] Clayton D (1999). A generalization of the transmission/disequilibrium test for uncertain-haplotype transmission. Am J Hum Genet.

[pgen-0030192-b058] Barrett JC, Fry B, Maller J, Daly MJ (2005). Haploview: analysis and visualization of LD and haplotype maps. Bioinformatics.

[pgen-0030192-b059] Purcell S, Neale B, Todd-Brown K, Thomas L, Ferreira MAR (2007). PLINK: a toolset for whole-genome association and population-based linkage analysis. Am J Hum Genet.

[pgen-0030192-b060] Purcell S, Daly MJ, Sham PC (2007). WHAP: haplotype-based association analysis. Bioinformatics.

[pgen-0030192-b061] Excoffier L, Slatkin M (1995). Maximum-likelihood estimation of molecular haplotype frequencies in a diploid population. Mol Biol Evol.

[pgen-0030192-b062] Fry B (2005). Computational Information Design.

[pgen-0030192-b063] The International HapMap Consortium (2005). A haplotype map of the human genome. Nature.

