# Supplementary material for: Identification of Two Independent Risk Factors for Lupus within the MHC in United Kingdom Families
Source: PLoS Genet. 2007 Nov 9;3(11):e192. doi: 10.1371/journal.pgen.0030192 (PMC2065882; doi:10.1371/journal.pgen.0030192)
Supplement: Supplementary file 1 [file pgen.0030192.sg001.pdf]

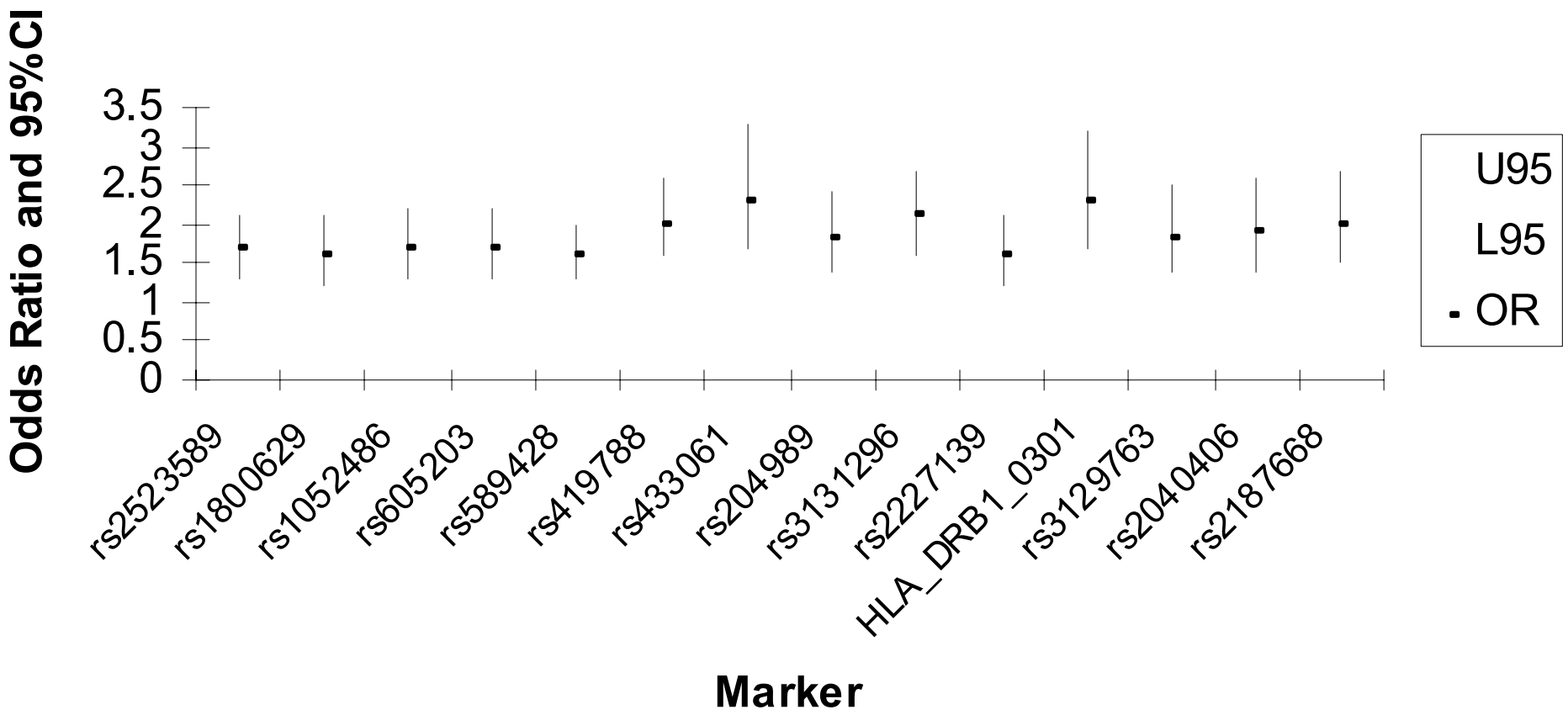
Odds Ratio and 95%CI
3.5
3
2.5
2
1.5
1
0.5
0
rs2523589
rs1800629
rs1052486
rs605203
rs589428
rs419788
rs433061
rs204989
rs3131296
rs2227139
HLA_DRB1_0301
rs3129763
rs2040406
rs2187668
Marker
U95
L95
OR
