# Supplementary material for: Identification of Two Independent Risk Factors for Lupus within the MHC in United Kingdom Families
Source: PLoS Genet. 2007 Nov 9;3(11):e192. doi: 10.1371/journal.pgen.0030192 (PMC2065882; doi:10.1371/journal.pgen.0030192)
Supplement: Supplementary file 2 [file pgen.0030192.sg002.pdf]

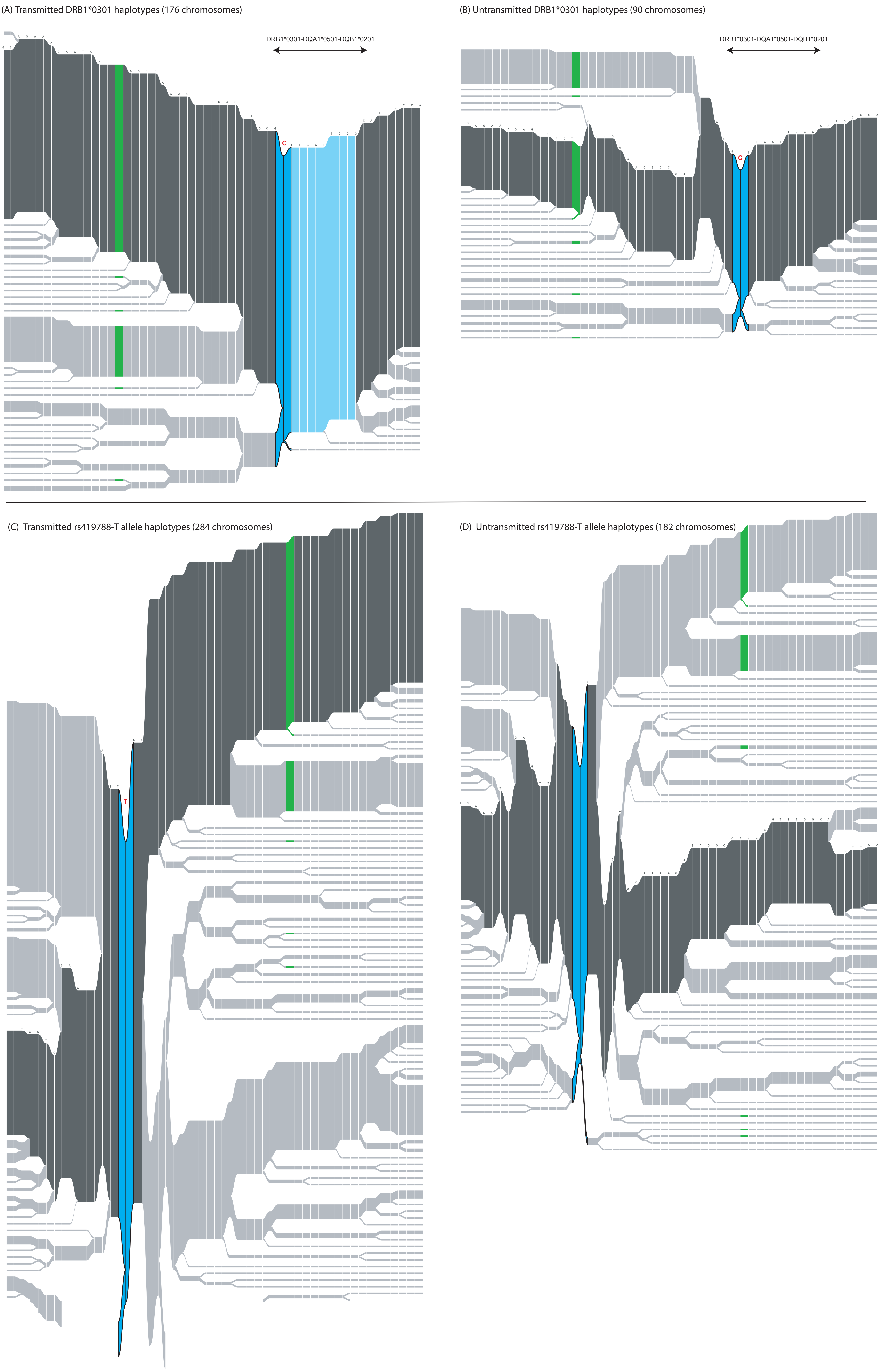

(A) Transmitted DRB1*0301 haplotypes (176 chromosomes)
DRB1*0301-DQA1*0501-DQB1*0201
C
(B) Untransmitted DRB1*0301 haplotypes (90 chromosomes)
DRB1*0301-DQA1*0501-DQB1*0201
C
(C) Transmitted rs419788-T allele haplotypes (284 chromosomes)
T
(D) Untransmitted rs419788-T allele haplotypes (182 chromosomes)
T
