# Supplementary material for: Identification of Two Independent Risk Factors for Lupus within the MHC in United Kingdom Families
Source: PLoS Genet. 2007 Nov 9;3(11):e192. doi: 10.1371/journal.pgen.0030192 (PMC2065882; doi:10.1371/journal.pgen.0030192)
Supplement: Supplementary file 3 [file pgen.0030192.st004.doc]

| **DRB1 genotype** | **Renal** | **Anti-C1q** | **ACLG** | **ACLM** | **Anti-Ro** | **Anti-La** | **Anti-RNP** | **Anti-Sm** |
| --- | --- | --- | --- | --- | --- | --- | --- | --- |
| **Total n=314** | 36 (112/314) | 14 (44/314) | 27 (83/312) | 12 (38/311) | 31 (93/305) | 8 (24/304) | 11 (34/305) | 4 (12/299) |
| **Non-0301**  **n=151** | 31 (46/151) | 15 (22/151) | 24 (36/150) | 11 (16/149) | 25 (36/145) | 1.4 (2/145) | 14 (20/145) | 6 (8/141) |
| **0301 HOM**  **n=20** | 45 (9/20) | 20 (4/20) | 20 (4/20) | 20 (4/20) | 35 (7/20) | 30 (6/20)^†^ | 10 (2/20) | 5 (1/20) |
| **0301 HET**  **n=127** | 40 (51/127) | 13 (16/127) | 29 (37/126) | 12 (15/126) | 39 (48/124)* | 13 (16/123)^†^ | 8 (18/124) | 2.5 (3/122) |
| **0301 HOM+HET**  **n=147** | 41 (60/147) | 14 (20/147) | 28 (41/146) | 13 (19/146) | 38 (55/144)* | 15 (22/143)^†^ | 8 (12/144) | 3 (4/142) |

**Table S4. Association of *HLA-DRB1*0301* genotypes with SLE subphenotypes in 314 UK SLE probands**

NOTE. - The data is presented as a percentage of UK SLE probands positive for the particular subphenotype followed by the actual genotype counts in parentheses. The Chi-square test was used to compare *HLA-DRB1*0301* genotypes with the presence or absence of each subphenotype. *nominal p<0.025, corrected p = 0.8 for 32 tests; ^†^nominal p<0.001, corrected p = 0.03 for 32 tests. Corrected p values were calculated using the overconservative Bonferroni method where corrected p = nominal p x number of tests.
